# Predictors for use of psychosocial services in patients with metastatic colorectal cancer receiving first line systemic treatment

**DOI:** 10.1186/s12885-019-5318-9

**Published:** 2019-02-01

**Authors:** Claudia S. E. W. Schuurhuizen, Annemarie M. J. Braamse, Inge R. H. M. Konings, Henk M. W. Verheul, Joost Dekker

**Affiliations:** 1 Department of Medical Oncology, VU University medical center, Cancer Center Amsterdam, Amsterdam, the Netherlands; 2 Department of Psychiatry and Amsterdam Public Health research institute, VU University Medical Center, Amsterdam, the Netherlands; 3 Department of Medical Psychology and Amsterdam Public Health Institute, Academic Medical Center, Cancer Center Amsterdam, Amsterdam, the Netherlands

**Keywords:** Metastatic colorectal cancer, Palliative treatment, Psychosocial services, Predictors

## Abstract

**Background:**

Patients with advanced disease experience high levels of psychological distress, yet there is low uptake of psychosocial services offered to patients who screened positive for distress. In this study we aimed to identify predictors for use of psychosocial services in patients with metastatic colorectal cancer (mCRC) receiving first line chemotherapy enrolled in a prospective cluster randomized trial (CRT).

**Methods:**

Patients completed measures on psychological distress, physical distress, and quality of life at baseline. Demographics, clinical characteristics at baseline and clinical events during treatment (e.g. severe adverse events, clinical benefit) were extracted from patient records. Patients reported psychosocial service utilization in- and outside the hospital after 10, 24 and 48 weeks of treatment. Multivariable logistic regression models were used to identify predictors for the use of psychosocial services.

**Results:**

Out of 349 patients, seventy patients (20.0%) used psychosocial support services during the follow-up period. Use of psychosocial services was associated with younger age, a higher educational level, presence of more pain (at baseline), and the expressed need to talk to a professional (at baseline). In addition, patients without progressive disease within the first ten weeks of treatment were more likely to use psychosocial services .

**Conclusions:**

One in five patients with mCRC receiving first line palliative treatment used psychosocial services during this prospective longitudinal CRT. Sociodemographic factors (age, education), clinical factors (pain and no progressive disease) and the expressed need to talk to a professional predicted use of psychosocial services. Identification of these predictors may contribute to the understanding of factors that determine the need for psychosocial services.

**Trial registration:**

Netherlands Trial Register NTR4034.

## Background

Over the past decades, research has demonstrated the considerable emotional impact that colorectal cancer (CRC) and its treatment can have on patients [1]. Self-reported psychological distress occurs in approximately one third of CRC patients [2–5]. Patients with advanced disease seem to be even more susceptible to develop symptoms of psychological distress [6]. A higher level of psychological distress is associated with lower quality of life (QOL), non-adherence to therapy, and poorer prognosis [7–9]. A variety of psychological interventions have been shown to be effective in reducing symptoms of distress and improving QOL [10]. As a result, current guidelines for cancer care have recommended to routinely screen for psychological distress and to subsequently offer treatment to those scoring above the cut off for distress [11].

Yet, the majority of patients scoring above the cutoff for distress decline professional intervention [12–15]. It seems that the presence of psychological distress does not necessarily equate to the need of patients for psychosocial support [2, 12, 16–18]. Distress as such does not seem to be a sufficient reason to use psychosocial services, as many patients scoring above the cut off for distress decline these services. Therefore, instead of assuming that all distressed patients need psychosocial services, it seems important to take one step back and to compare patients who use services against those who do not. Knowledge on factors related to the actual use of psychosocial services may contribute to our understanding of the factors that determine the need for psychosocial services. We aimed to identify predictors for use of psychosocial services in patients with mCRC receiving first line treatment. Potential predictors included demographic, clinical, and psychosocial factors at baseline and clinical factors during treatment.

## Methods

This paper reports on a secondary analysis on data obtained in the TES trial (Targeted selection, Enhanced care, Stepped care trial). In this trial the effectiveness of a combined screening and treatment program to improve psychological distress compared with care as usual was evaluated (Netherlands Trial Register (NTR4034) [19]. The study was approved by the Medical Ethics Committee of VU University Medical Center (2013/10). Analyses are based on 349 outpatients with a confirmed diagnosis of mCRC starting first line systemic treatment who met the eligibility criteria, recruited throughout 16 hospitals in the Netherlands, between July 2013 and October 2016. All patients provided written informed consent. A description of the study protocol and results of the original trial have been published previously [14, 19]. In brief, there were no significant differences in course of distress between the treatment arms. Further, no differences in recognition and management of distress or use of psychosocial support between groups were found, although satisfaction with care and cognitive functioning were significantly better in the intervention group.

### Outcome and measurements

Demographic and clinical characteristics were obtained from patients and medical charts at baseline, i.e. start of first line systemic treatment. In addition, during the 48 weeks of the TES study, information on clinical events was collected. At baseline, patient-reported psychological characteristics were measured with the following tools. The HADS (Hospital Anxiety and Depression Scale) and DT/PL (Distress Thermometer and corresponding Problem List) were used to assess distress and the need for referral with a recall period of two weeks, respectively. The HADS consist of 14 questions with scores ranging from 0 to 42, the recommended cut off score for distress in cancer patients is ≥13 [20]. The DT/PL comprises of the Distress Thermometer ranging from 0 to 10, followed by a list of problems evaluating a wide range of problems and a single question evaluating the need to talk to a professional [2]. A score of 5 or higher on the DT has been shown to indicate presence of distress [2]. QOL was measured by the EORTC-QLQ-C30 version 3.0 [21], consisting of five functional scales (physical, role, cognitive, emotional, and social), symptom scales and individual symptom items, and perceived financial impact of the disease.

During treatment, the following clinical events were assessed by clinicians: treatment modification or discontinuation, clinical benefit during the first 10 weeks of treatment (progression vs stable disease or partial response), occurrence of severe (i.e. grade III/IV) adverse events (AEs, these were graded and assigned by clinicians using the National Cancer Institute’s Common Terminology Criteria for Adverse Events (NCI-CTCAE) as mild (Grade 1), moderate (Grade 2), severe (Grade 3), or life-threatening (Grade 4) [22]) and total number of unscheduled admissions. Psychosocial service utilization in- and outside the hospital was assessed using an adapted version of the TiC-P questionnaire [23] after 10, 24 and 48 weeks of treatment with a recall period of three months. The TiC-P is a feasible and reliable instrument for collecting data on medical consumption and productivity losses [23, 24]. The first part of the TiC-P includes 14 structured yes/no questions on relevant medical resource items each followed by a question on the volume of medical consumption. The questions include contacts within the mental healthcare sector (regional mental healthcare organization, psychiatrist/psychologist or psychotherapist in private practices or outpatient hospital, institutional day-care treatment, Consultation Agency for Alcohol and Drug addiction (CAD), self-help group) and contacts with general healthcare providers (general practitioner, allied health professional, social worker, providers of alternative medicine, outpatients visits to medical specialists, hospital admission and contacts with a company doctor) and the use of medication. For the current analyses we evaluated contacts with social workers, contacts with services in regional mental healthcare organizations, contacts with psychiatrists/psychologists in private practice, and contacts with psychiatrists/psychologist in hospital settings.

### Statistical analyses

Descriptive statistics were calculated to summarize all variables and to outline the pattern of use of psychosocial services in patients from the TES trial. Differences between patients who had used psychosocial services versus those who had not used psychosocial services during follow-up were computed using the chi-square test for categorical data and t-test for metric data. Two separate multivariable analyses were undertaken to identify predictors for the use of psychosocial support. In *the first analysis*, baseline variables potentially related to the use of psychosocial support were evaluated. Explanatory variables included gender (male vs female), age at randomization, married/living together vs living alone, education level (low: primary education, middle: lower general secondary education, intermediate vocational education or high school, high: higher vocational education or university), employment (working yes vs no), functional status (defined by the Eastern Cooperative Oncology Group Performance Status (ECOG PS), which is a score ranging from zero (“fully active”) through three (“capable of only limited self-care”) to five (“dead”) [25]), right or left sided tumor, number of prior cancer-related treatments, type of chemotherapy (capecitabine vs CAPOX vs FOLFOX or other), total number of (organs with) metastases, time since initial diagnosis in months (< 1.5 months vs 1.5–10 months vs > 10 months), time since diagnosis of metastatic disease in months (< 1.0 months vs 1.0–2.5 months vs > 2.5 months), comorbidity (number of comorbid conditions as reported by patients at baseline), allocation to treatment arm of the parent study (intervention vs control) and participation in other clinical studies (yes vs no), distress on HADS (score of < 13 or ≥ 13), distress score on DT (< 5 or ≥ 5), number of physical problems reported on PL, number of emotional problems reported on PL, need to talk to a professional (yes vs no), scores on functional QOL scales, global QOL, fatigue and pain score on EORTC-QLQ-C30. Variables were included in the multivariable logistic regression equation if they had an univariable association (*p* ≤ .25) with the criterion [26]. A backward stepwise regression model was computed to select the final model that included predictive factors which were statistically significant at the 0.10 level. Variables not significantly contributing to the multivariable logistic regression equation were removed. In *the second analysis* it was evaluated whether clinical factors during treatment predict use of psychosocial support. Explanatory variables included clinical benefit during the first 10 weeks of chemotherapy treatment, number of severe AEs, any severe AE during treatment, any admission during treatment, and number of admissions. Using the final model on baseline predictors as a base, variables were included in this model if they had an univariable association (*p* ≤ .25) with the criterion. A backward stepwise regression model was computed to select the final model that included predictive factors which were statistically significant at the 0.10 level. Variables not significantly contributing to the multivariable logistic regression equation were removed. The explained variance of the model was assessed by use of Nagelkerke’s *R*^2^. Since multicollinearity can influence the estimated beta parameter, the variable inflation factor (VIF) for each variable included in the regression equations was calculated (i.e. a VIF greater than 2.5 indicates multicollinearity) [27, 28]. Data were analyzed using IBM SPSS statistics version 22.0 (IBM Corp., Armonk, NY).

## Results

The sample consisted of all 349 patients enrolled in the TES study [14]. Table 1 contains sample characteristics. The 349 patients ranged in age from 23 to 83 years at diagnosis (mean age 66.1 ± 10.2 years); 224 (64.2%) were male, 256 (73.4%) were married/living with a partner, 93 (26.7%) scored above the cutoff for distress (measured with the HADS), and 34 (9.7%) expressed a need to talk to a professional. At the time of baseline assessment all patients were receiving chemotherapy, 72 patients (20.6%) monotherapy, 274 (78.5%) an oxaliplatin-based combination regimen; for 3 patients type of chemotherapy was unknown. All treatments were administered with palliative intent. Seventy (20.0%) out of 349 patients made use of psychosocial services during the 48-week follow-up period. Of these, 36 (51.4%) had contact with social workers, 6 (8.6%) had contact with services in regional mental healthcare organizations, 13 (18.6%) had contact with psychiatrists/psychologists in private practices, 4 (5.7%) had contact with psychiatrists/psychologists in the hospital setting and the remaining 27 patients (38.6%) had contact with multiple psychosocial services. Twenty-six of 93 (28.4%) patients who scored above the cutoff for distress on the HADS at baseline used psychosocial services during the 48-week follow-up period. Twelve of 34 (35.3%) patients who had expressed a need to talk to a professional, used psychosocial services during the 48-week follow-up period.

**Table 1:** Results of univariable analyses on demographic, clinical and psychosocial baseline variables

	No use of psychosocial support services (*n* = 279)(n,%)	Use of psychosocial support services (*n* = 70)(n,%)	*p-value*
Age, years			*0.003
Mean age (SD)	66.92 (9.7)	62.74 (11.41)	
Gender			0.415
Male	182 (65.2)	42 (60.0)	
Female	97 (34.8)	28 (40.0)	
Treatment arm in TES trial			0.610
Intervention	149 (53.4)	35 (50)	
Control	130 (46.6)	35 (50)	
ECOG PS^a^			0.714
0	67 (24.0)	15 (21.4)	
1	87 (31.2)	18 (25.7)	
2	8 (2.9)	3 (4.3)	
Missing	117 (41.9)	34 (48.6)	
Primary tumor location			0.695
Right-sided	84 (30.1)	23 (32.9)	
Left-sided	192 (68.8)	47 (67.1)	
Missing	3 (1.1)	–	
Chemotherapy regimen			0.875
Capecitabine	59 (21.1)	13 (18.6)	
CAPOX	190 (68.1)	50 (71.4)	
FOLFOX/Other	27 (9.7)	7 (10.0)	
Missing	3 (1.1)	–	
Number of comorbidities			0.980
Mean (SD)	2.33 (1.78)	2.33 (2.00)	
Marital status			0.369
Married/domestic partnership	207 (74.2)	49 (70.0)	
Unmarried/divorced/widowed	68 (24.4)	21 (30.0)	
Missing	4 (1.4)	–	
Number of persons in household			0.615
1	49 (17.6)	13 (18.6)	
2	177 (63.4)	42 (60.0)	
3	23 (8.2)	4 (5.7)	
> 3	26 (9.3)	11 (15.7)	
Missing	4 (1.4)	–	
Education^b^			*0.041
Low	17 (6.1)	2 (2.9)	
Middle	184 (65.9)	39 (55.7)	
High	72 (25.8)	29 (41.4)	
Missing	6 (2.2)	0 (0)	
Currently working			0.949
Yes	65 (23.3)	16 (22.9)	
No/retired	210 (75.3)	54 (77.1)	
Missing	4 (1.4)	–	
Time from diagnosis primary tumor until start study			0.848
< 1.5 months	94 (33.7)	22 (31.4)	
1.5–10 months	89 (31.9)	25 (35.7)	
> 10 months	93 (33.3)	23 (32.9)	
Missing	3 (1.1)	–	
Time from diagnosis metastatic disease until start study			0.666
< 1.0 months	95 (34.1)	27 (38.6)	
1.0–2.5 months	87 (31.2)	23 (32.9)	
> 2.5 months	94 (33.7)	20 (28.6)	
Missing	3 (1.1)	–	
Prior cancer related treatment			
No	85 (30.5)	23 (32.9)	0.699
Yes	194 (69.5	47 (67.1)	
Prior treatment metastases			
No	214 (76.7)	56 (80.0)	0.657
Yes	62 (22.2)	14 (20.0)	
Missing	3 (1.1)	–	
Number of organs with metastases			0.599
Median	2	2	
Total number of metastases			
Median	11.5	13.0	0.877
Participation in other trials			0.718
No	170 (60.9)	41 (58.6)	
Yes	109 (39.1)	29 (41.4)	
HADS			
Total score	9.10 (6.30)	11.17 (7.47)	*0.020
Distress on HADS No	208 (74.6)	44 (62.9)	*0.033
Yes	67 (24.0)	26 (37.1)	
Missing	4 (1.4)	–	
DT/PL			
DT score	4.33 (2.68)	4.75 (2.40)	*0.242
Total of physical problems	5.78 (6.29)	7.37 (4.61)	*0.020
Total of emotional problems	1.95 (2.11)	2.74 (2.57)	*0.009
Need to talk to professional No	257 (92.1)	58 (82.9)	*0.023
Yes	22 (7.9)	12 (17.1)	
Quality of life (QLQ-C30)			
Physical functioning	74.84 (20.30)	72.76 (21.88)	0.451
Role functioning	65.69 (30.70)	58.81 (30.13)	*0.010
Emotional functioning	78.25 (18.12)	72.86 (21.41)	*0.095
Cognitive functioning	89.44 (14.97)	86.90 (16.76)	*0.219
Social functioning	78.08 (23.89)	69.05 (31.38)	*0.010
Global QOL	63.77 (21.73)	60.00 (21.78)	*0.197
Pain score	16.00 (23.75)	40.15 (26.57)	*0.174
Fatigue score	25.00 (16.67)	45.08 (44.44)	*0.009

*Abbreviations*: *SD* standard deviation, *ECOG PS* Eastern Cooperative Oncology Group Performance Status, *HADS* Hospital Anxiety and Depression Scale, *DT/PL* Distress Thermometer and Problem list, *QLQ-C30* Quality of Life Questionnaire-C30. ^*^Characteristics were included in the multivariate model if they had an univariable association (*p* ≤ .25). ^a^ECOG PS 0: Fully active, able to carry on all pre-disease performance without restriction. ECOG PS 1: Restricted in physically strenuous activity but ambulatory and able to carry out work of a light or sedentary nature, e.g., light house work, office work. ECOG PS 2: Ambulatory and capable of all selfcare but unable to carry out any work activities; up and about more than 50% of waking hours. ^b^Low: primary education, middle: lower general secondary education, intermediate vocational education or high school, high: higher vocational education or university

Table 1 contains the univariable analyses for baseline sociodemographic, clinical and psychological factors and their associations with the use of psychosocial support. Patients in the group that used psychosocial services were significantly younger at baseline and were more often higher educated. In addition, they reported higher levels of distress and were more frequently classified as being distressed (i.e. a score of ≥13 on HADS), reported more emotional and physical problems, and expressed the need to talk to a professional more frequently. Finally, patients who used psychosocial services demonstrated lower levels of role and social functioning at baseline and were significantly more fatigued. There were no differences for gender, marital status, employment, tumor burden or ECOG PS at baseline. Table 2 contains the univariable analyses for clinical events during follow-up and their associations with the use of psychosocial support. Patients in the group that used psychosocial services were more often responding to therapy within the first ten weeks of treatment (i.e. stable disease or partial response), whereas patients who did not use psychosocial services more often had had progression of disease within the first ten weeks of treatment.

**Table 2:** Univariable analyses for clinical variables during treatment predictive for the use of psychosocial support services

	No use of psychosocial support services (n = 279)(n,%)	Use of psychosocial support services (n = 70)(n,%)	*p-value*
Progression of disease within first 10 weeks of treatment			*0.089
No	242 (86.7)	66 (94.3)	
Yes	37 (13.3)	4 (5.7)	
Progression of disease during follow up			0.289
No	93 (33.3)	28 (40.0)	
Yes	186 (66.7)	42 (60.0)	
Total number of grade 3–4 AEs during 48 weeks follow up	1.64 (2.12)	2.06 (2.65)	*0.166
Total number of admissions during 48 weeks follow up	1.79 (1.34)	2.13 (1.98)	*0.216

*Abbreviations*: *AEs* adverse events. ^*^Characteristics were included in the multivariate model if they had an univariable association (*p* ≤ .25)

Table 3 contains the regression parameters for the baseline predictors retained in the backward logistic regression analysis. Use of psychosocial services was associated with younger age, presence of more pain (at baseline), an expressed need to talk to a professional and a higher educational level. The final model with baseline characteristics had an overall classification rate of 81.2% with Nagelkerke *R*^*2*^ statistic of 0.118. In Table 4 the final model with baseline variables was used as a base and contains the regression parameters for the variables retained in the backward logistic regression analysis after adding clinical factors during treatment. Combined with the four baseline variables of the first model, patients who had progressive disease within the first ten weeks of treatment were *less* likely to use psychosocial services. The final model combining selected baseline variables and clinical variables over time had an overall classification rate of 81.5% with Nagelkerke *R*^*2*^ statistic of 0.140.

**Table 3:** Final multivariable model of baseline predictors

Predictive factor^a^	Wald	*p*-value	Odds Ratio	95% C.I.for EXP(B)		VIF
				Lower	Upper	
Need to talk to professional	4.451	.035	2.360	1.063	5.240	1.010
Pain score at baseline	8.053	.005	1.015	1.005	1.025	1.009
Age	8.066	.005	.963	.938	.988	1.022
Education	5.823	.054				1.031
Middle compared to low	.084	.772	1.255	.270	5.837	
High compared to low	1.340	.247	2.518	.527	12.024	

*Abbreviations*: *OR* odds ratio, *VIF* variable inflation factor. ^a^Predictive factors were statistically significant at the least at the 0.10 level

**Table 4:** Final multivariable model of baseline predictors and clinical predictors

Predictive factor^a^	Wald	*p*-value	Odds Ratio	95% C.I.for EXP(B)		VIF
				Lower	Upper	
Need to talk to professional	4.712	.030	2.429	1.090	5.412	1.010
Pain score at baseline	9.895	.002	1.017	1.006	1.028	1.030
Age	6.938	.008	.965	.940	.991	1.030
Education	6.190	.045				1.033
Middle compared to low	.128	.720	1.327	.283	6.229	
High compared to low	1.558	.212	2.729	.564	13.204	
Progression within first 10 weeks of treatment	4.136	.042	.314	.103	.959	1.029

*Abbreviations*: *OR* odds ratio, *VIF* variable inflation factor. ^a^Predictive factors were statistically significant at the least at the 0.10 level

## Discussion

This study prospectively investigated the predictors for use of psychosocial services in patients with mCRC receiving first line palliative treatment. Psychosocial support was broadly defined as contacts with social workers, contacts with services in regional mental healthcare organizations, contacts with psychiatrists/psychologists in private practice, and contacts with psychiatrists/psychologist in hospital settings. Our approach is unique as we assessed and distinguished possible predictors at baseline and clinical predictors during treatment. In line with previously reported results [16], approximately one in five patients used psychosocial services at any point during their 48-weeks clinical follow-up. In this sample of patients with mCRC, those patients having a younger age, having completed higher education levels, indicating higher levels of pain and expressing the need to talk to a professional were found to be more likely to use psychosocial services. Furthermore, absence of disease progression within the first ten weeks of treatment was found to be a clinical predictor for the use of these services.

The relationship between younger age and the use of psychosocial services is consistent with previous research [29–31]. A plausible explanation could be that advanced disease may be more traumatic and require greater life adjustments in younger patients who are in a different phase of life with work, children and relationships, compared to older patients [28, 31]. In addition, younger patients may be more aware of psychosocial services [28]. Users of psychosocial support in our study were more likely to have completed higher levels of education, a trend that is consistent with the literature [30, 32]. A knowledge gap may exist between patients with different education levels, those with less education may have greater information needs regarding the benefits and availability of psychosocial services [32]. In line with findings from O’Hea et al. [29], patients reporting more pain were seen to use psychosocial services more often. In agreement with our findings that showed that the expressed need to talk to a professional was a predictor for use of psychosocial services, asking patients about their desire for help to highlight who are willing to accept psychosocial interventions has been proposed before as an efficient way of connecting patients to psychosocial services [16, 33].

The lack of association between gender and marital status and of use of psychosocial services contrasts with previous studies [28, 29, 31, 34], in which female and single patients have been shown to be using psychosocial services more often. However, those results were obtained mainly in heterogeneous study samples, predominated by younger, female patients suffering from breast cancer. Our study involved a homogeneous sample of on average older, mostly male mCRC patients, whose emotional concerns and needs, or access to psychosocial care services may have been different.

Absence of disease progression was associated with higher use of psychosocial services, while disease progression itself has previously been shown to be an important predictor for psychological distress [14, 35]. One hypothesis could be that patients’ focus when facing disease progression may shift towards end-of-life issues, in which energy is more likely to be spent on preparation for death and achieving a sense of completion than in using psychosocial support services [36, 37].

Other clinical factors, such as burden of disease, time since diagnosis (of metastasis), type of systemic treatment, or amount of suffered high grade AEs and subsequent admissions did not appear to be predictors. Low predictive associations between clinical variables and need for psychological support services were demonstrated before [38]. These combined findings suggest that clinical (both pre- and on-treatment) variables are largely not predictive for use of psychosocial support, and emphasize that patient sociodemographic characteristics and self-report assessments of patients’ physical and psychosocial well-being are essential in predicting which patients will use psychosocial services.

In line with previous work [34, 38], our results demonstrated that out of 93 patients who scored above the cutoff for distress at baseline, fewer than one third used psychosocial services. It has been demonstrated that the majority of patients show strong resilience following life-threatening events, characterized by transient symptoms of distress and return to normal levels without need for psychosocial interventions [39, 40]. This kind of emotional distress may even facilitate adaptation to cancer [12]. A minority of distressed patients does not seem to be able to cope with emotions related to cancer; this group of patients may be in need for referral to specialized mental healthcare [12].

Even though expressing the need to talk to professionals was found to be predictive for use of psychosocial services, out of 34 patients that expressed the subjective need to talk to a professional, services were only used by 12 (35.3%) of these patients. This latter finding is concerning. In our study we did not further investigate why patients with an expressed need did or did not use support services. Known barriers to use of psychosocial services are lack of knowledge, financial constraints, lack of access, as well as lack of confidence in and negative perceptions towards psychosocial services [18, 41]. In order for those patients with a need for support to actually access these services, future studies should focus on the promotion of psychosocial support services [42]. However, it is worth mentioning that the item used to assess the need to talk to a professional could have been interpreted differently by patients, as there was no specific reference to mental health and this need may have referred to various disciplines. In a recently updated version of the Distress Thermometer, patients can now indicate with whom they would like to talk followed by a list of both physical and psychosocial healthcare providers [43].

### Study limitations

Limitations of the present study include the self-reporting of use of psychosocial services, that may have under- or overestimated the actual use of these care services. Self-reported data are argued to be less reliable and threatened by bias arising from social desirability and recall period [44]. Second, this study did not make a clear distinction between type of psychosocial needs and problems. For some needs, especially needs resulting from practical or logistical issues, psychosocial services may not be appropriate. In addition, the analyses were conducted on a homogenous sample of particularly middle-aged, male, patients with advanced CRC participating in a CRT. Further research is needed to determine whether these findings apply to community-based settings as well and whether these findings are generalizable to younger or older patients. Furthermore, the need for psychosocial support was assessed with one single item question only. Another limitation was the sample size. Even though a total of 349 patients participated in the original trial, only 70 made use of psychosocial support. As a consequence some of the subgroups only contained a small number of patients, which could limit the generalizability of the results, warranting external validation.

### Clinical implications

Our results can be used to inform clinicians in intervention practices as these provide insight into characteristics of patients receptive to psychosocial care. Identification of predictors for use of psychosocial services in patients with mCRC may allow resources to be directed more efficiently to patients who need and want help. In addition, our study findings underscore that relying on screening for distress to identify patients that use psychosocial services is less successful than has been assumed [33]: the need to talk to a professional, instead of the level of distress, predicted use of psychosocial services. Instead of relying on screening for distress, psychosocial services could be targeted to patients expressing the need to talk to a professional. This could be supplemented with more targeted assessments of patients at risk for psychosocial problems [45]. In addition, it should be emphasized that there may be a group of individuals at risk of suffering from negative psychosocial outcomes that is unlikely to use psychosocial services (e.g. less educated, older man), future research should focus on potential barriers to this use and on novel targeting tools to include these patients that my benefit from support.

## Conclusions

To conclude, approximately 20% of patients with mCRC starting with first line palliative treatment used psychosocial services during 48 weeks of follow up. The following predictors for use of psychosocial support were identified: being younger, reporting higher levels of pain, being higher educated, expressing a need to talk to a professional and the absence of progressive disease within the first weeks of treatment. These findings contribute to the understanding of factors that determine the need for psychosocial services.

## Abbreviations

AEs: Adverse events
CAD: Consultation Agency for Alcohol and Drug addiction
CRC: Colorectal cancer
CRT: Cluster randomized controlled trial
DT: Distress Thermometer
DT/PL: Distress Thermometer / Problem List
ECOG PS: Eastern Cooperative Oncology Group Performance Status
HADS: Hospital Anxiety and Depression Scale
mCRC: metastatic colorectal cancer
NCI-CTCAE: National Cancer Institute’s Common Terminology Criteria for Adverse Events
QOL: Quality of life
TES: Targeted selection, enhanced and stepped care
VIF: Variable inflation factor
